# Selection, affinity maturation, and characterization of a human scFv antibody against CEA protein

**DOI:** 10.1186/1471-2407-6-41

**Published:** 2006-02-24

**Authors:** Emiliano Pavoni, Michela Flego, Maria Luisa Dupuis, Stefano Barca, Fiorella Petronzelli, Anna Maria Anastasi, Valeria D'Alessio, Angela Pelliccia, Paola Vaccaro, Giorgia Monteriù, Alessandro Ascione, Rita De Santis, Franco Felici, Maurizio Cianfriglia, Olga Minenkova

**Affiliations:** 1 Kenton Labs, c/o Sigma-Tau, via Pontina, km 30.400, 00040 Pomezia (RM), Italy; 2 Pharamcogenetics, Drug Resistance & Experimental Therapeutics Section, Department of Drug Research and Evaluation of Italian National Institute of Health (Istituto Superiore di Sanità), Viale R. Elena 299, 00161 Rome, Italy; 3 Immunology Department, Sigma-Tau, via Pontina, km 30.400, 00040 Pomezia (RM), Italy; 4 Department of Microbiology, Genetics, Molecular Biology, University of Messina, Messina, 98100, Italy

## Abstract

**Background:**

CEA is a tumor-associated antigen abundantly expressed on several cancer types, including those naturally refractory to chemotherapy. The selection and characterization of human anti-CEA single-chain antibody fragments (scFv) is a first step toward the construction of new anticancer monoclonal antibodies designed for optimal blood clearance and tumor penetration.

**Methods:**

The human MA39 scFv, selected for its ability to recognize a CEA epitope expressed on human colon carcinomas, was first isolated from a large semi-synthetic ETH-2 antibody phage library, panned on human purified CEA protein. Subsequently, by *in vitro *mutagenesis of a gene encoding for the scFv MA39, a new library was established, and new scFv antibodies with improved affinity towards the CEA cognate epitope were selected and characterized.

**Results:**

The scFv MA39 antibody was affinity-maturated by *in vitro *mutagenesis and the new scFv clone, E8, was isolated, typed for CEA family member recognition and its CEACAM1, 3 and 5 shared epitope characterized for expression in a large panel of human normal and tumor tissues and cells.

**Conclusion:**

The binding affinity of the scFv E8 is in a range for efficient, *in vivo*, antigen capture in tumor cells expressing a shared epitope of the CEACAM1, 3 and 5 proteins. This new immunoreagent meets all criteria for a potential anticancer compound: it is human, hence poorly or not at all immunogenic, and it binds selectively and with good affinity to the CEA epitope expressed by metastatic melanoma and colon and lung carcinomas. Furthermore, its small molecular size should provide for efficient tissue penetration, yet give rapid plasma clearance.

## Background

The construction of libraries of recombinant antibody fragments that are displayed on the surface of filamentous phage, and the selection of phage antibodies against target antigens, have become an important technological tool in generating new monoclonal antibodies for research and clinical applications. Human antibodies obtained by this method do not induce harmful immune response in patients, in comparison with murine monoclonal antibodies produced by the classic hybridoma techniques. Moreover, the affinity of selected antibodies can be improved through construction of mutant antibody libraries giving clones with greater affinity. Thus, naïve or semi-synthetic human antibody libraries can be used in the search for specific antibodies without immunization with respective antigens.

The carcinoembryonic antigen (CEA) is an oncofetal glycoprotein, containing 50% carbohydrate with a molecular weight of approximately 200 kDa [1], overexpressed in several tumor types of epithelial origin and known as an important and extensively used clinical tumor marker for colorectal and other carcinomas [2-4]. In tumor patients, with growth of the tumor mass, more CEA is accumulated in the blood. Therefore, the CEA serum levels can be used to monitor recurrent disease in the post-surgical surveillance of colorectal cancer [5], but not to localize the lesions.

The use of labeled monoclonal antibodies that target a tumor-associated antigen was first described by Goldenberg [6]. In several recent studies, anti-CEA antibodies were used to localize CEA-expressing solid tumors. Murine (CEA)-specific monoclonal antibodies were successfully tested in animals for their capacity to accurately localize tumors formed by human colorectal carcinoma cell lines with various levels of CEA expression and, as a result, their use is foreseen in radioimmunoguided surgery (RIGS) [7]. The immunoscintigraphy analysis, with an anti-CEA monoclonal antibody fragment labeled with ^99m^Tc, in patients with colorectal carcinoma recurrence, was evaluated as an effective method for early detection of pelvic and extrahepatic abdominal metastases [8]. A Phase I clinical trial was reported for RIGS with recombinant phage-selected scFv anti-CEA antibody fragment to CEA [9]. Rapid blood clearance, good tumor penetration, the lack of significant toxicity, and relatively simple production in bacteria, make such antibodies suitable for clinical practice.

We describe in this paper the selection of a new anti-CEA single-chain antibody fragment, MA39, from an ETH-2 synthetic antibody library [10]. This antibody recognizes human CEA protein in ELISA, Western blot and flow cytometry. The single-chain antibody was affinity-maturated by mutagenesis *in vitro*. The dissociation constant of the new maturated anti-CEA E8 antibody (1.39 × 10^-8^) was decreased more than 10-fold compared to the original MA39 (1.71 × 10^-7^). The specificity of E8 scFv was assessed by flow cytometry on a broad panel of human cells and BOSC23 cells, ectopically expressing members of the CEACAM protein family, and by immunohistochemical staining of tumor and normal human tissues.

## Methods

### Bacterial strains

Bacterial strains TG1 (*supE hsd*Δ5 *thi *Δ(*lac-proAB*) F' [*traD*36 *proAB*^+ ^*lacI*^*q*^*lacZ*ΔM15]) and DH5αF' (*supE44 *Δ*lac*U169 (*φ*80 *lacZ*ΔM15) *hsdR*17 *recA*1 *endA*1 *gyrA*96 *thi*-1 *relA*1 F' [*traD*36 *proAB*^+ ^*lacI*^*q*^*lacZ*ΔM15]) were used for phage antibody production. The HB2151 bacterial host strain (*nalr thi*-1 *ara *Δ(*lac-proAB*) F' [*proAB*^+ ^*lacI*^*q *^lacZΔM15]) was used for large-scale soluble antibody production.

### CEA biotinylation

One hundred μg of CEA (Sigma, St Louis, MO) were dissolved in 0.9 mL of PBS and incubated with 100 μL of biotinamidohexanoic acid N-hydroxysuccinimide ester (B2643, Sigma) at a concentration of 2.2 mg/mL in 2.8% DMSO at room temperature (RT); after 1 h incubation, 50 μL of 1 M NaHCO_3_, pH 9, were added to the protein. Sample was dialyzed against PBS overnight (ON) at 4°C.

### ETH-2 library and phage antibody selection

The ETH-2 synthetic human recombinant antibody library consists of a high array (more than 10^9 ^antibody combinations) of single-chain variable fragment antibodies (scFv) displayed on the surface of filamentous phage. It was built by random mutagenesis of the CDR3 of only three antibody gene germline segments (DP47 for the heavy chain, DPK22 and DPL16 for the light chain). The diversity of the heavy chain was created by randomizing four to six positions, replacing the pre-existing 95 to 98 positions of the CDR3. The diversity of the light chain was created by randomizing six positions (96 to 101) in the CDR3 of this chain (for details of ETH-2 library see [10]).

An aliquot of the ETH-2 library containing approximately 10^12 ^transducing units (TU) was incubated with 2.5 mL of 50 nM biotinylated CEA in 2% milk in PBS (MPBS) for 2 h at RT. The bound phage was captured by using streptavidin-coated M-280 Dynabeads (112.05, Dynal, Oslo, Norway) according to manufacturer's instructions, washed 15 times with TPBS (0.1% Tween 20 in PBS) and 15 times with PBS using a magnetic device. Bound phages were eluted with 500 μL of 100 mM triethylamine and the solution was immediately neutralized by adding 0.5 mL of 1 M Tris-HCl (pH 7.4). Eluted phages were used to infect *E.coli *TG1 cells and amplified for the next round of panning.

### Design and construction of first maturation library

**Figure 1:**
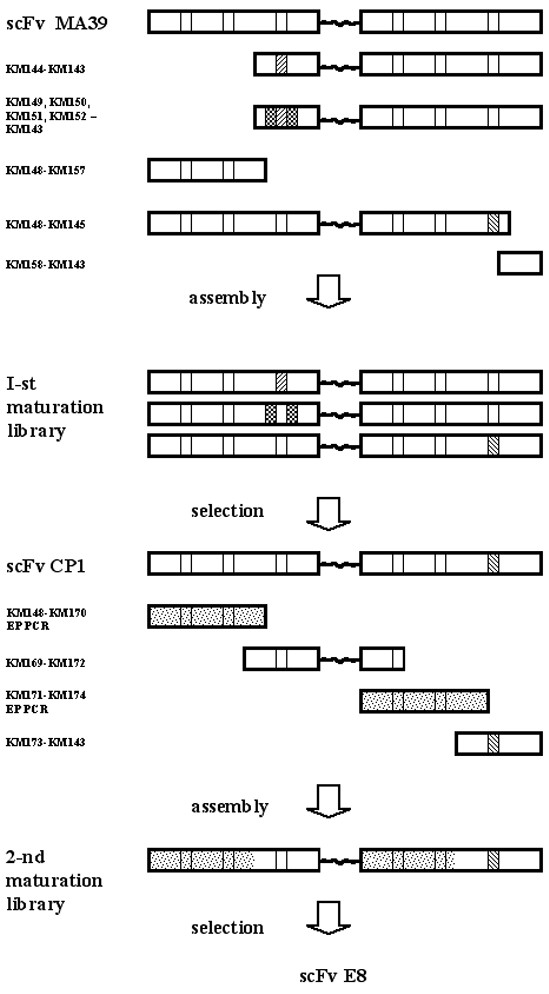
**Scheme of mutagenesis *in vitro***. First maturation library was generated by using partially degenerated oligonucleotides. Second maturation library was constructed by error-prone PCR method.

For the construction of MA39 mutated library, the gene of MA39 scFv clone selected from the ETH-2 library was used as template for PCR amplification (Figure 1). Mutated scFv gene fragments were generated by PCR amplification with primers KM144-KM143 and KM148-KM145 (Table 1), introducing random mutations in CDR3 regions of heavy or light chains with low frequency. The KM144 and KM145 oligonucleotides were synthesized with partial substitution by a four-nucleotide mixture on each position corresponding to CDR3 region with a frequency of 10% (central underlined part of the sequence) (MWG Biotech, Ebersberg, Germany). In addition, the antibody fragment containing CDR3 of the heavy chain was amplified using KM143 together with KM149, KM150, KM151 and KM152, including from two to three NNS random triplets at the 5' and 3' sites of CDR3-encoding sequence. Missing scFv antibody fragments were amplified with primers KM148-KM157 and KM158-KM143 for HC and LC, respectively. The oligonucleotides KM157 and KM158 were designed to obtain overlapping fragments allowing assembly of a full-length mutated scFv gene, as indicated in Figure 1. In each case, two resulting PCR fragments were agarose-purified and assembled by 5 cycles of PCR without primers and then amplified with external primers KM148-KM143. The final PCR product was agarose-purified, digested with restriction enzymes *Nco*I and *Not*I, and cloned into the plasmid of pDN332, containing FLAG tag sequence and a 6×His tag for protein purification [11], which was also digested with *Nco*I and *Not*I. A ligation mixture was used to transform the DH5αF'-competent cells to get an initial maturation library with a complexity of 2.5 × 10^6 ^scFv members.

### Second maturation library

For construction of CP1 mutated library, the gene of the CP1 scFv clone, selected from the first maturation library, was used as template for PCR amplification (Figure 1). CP1 clone was amplified with pairs of primers KM148-KM170, KM169-KM172, KM171-KM174 and KM173-KM143 (Table 1). Fragments amplified with underlined primers were mutated by error-prone PCR, introducing mutations in the corresponding fragment with a frequency of 1% to 2%. These primers were designed to amplify scFv gene regions corresponding to the CDR1 and CDR2 regions of both chains, as shown in Figure 1. PCR was performed in final volume 100 μL using Taq polymerase (Promega, Madison, WI) in presence of 6.5 mM MgCl_2_, 0.5 mM MnCl_2_, 1 mM dCTP, 1 mM dGTP, 1 mM dTTP, 0.2 mM dATP, and 0.1 mM dITP. The resulting four fragments were agarose gel-purified, and assembled to generate a full-length mutated scFv gene by five cycles of PCR-like amplification without primers. The KM143 and KM148 external primers were then added and the reaction was cycled another 25 times. The gel-purified PCR product was cut with *Nco*I and *Not*I, and ligated into the plasmid of pDN332, also digested with *Nco*I and *Not*I. The maturation library had a complexity of 1.7 × 10^7 ^scFv clones. After two rounds of selection, the phage pool was amplified and single-chain DNA was purified by PEG/NaCl precipitation and phenol extraction [12]. All procedures of mutagenesis, assembly and cloning were repeated, yielding a library with a complexity of 1.6 × 10^6 ^independent scFv clones.

### Correction of TGA codon for soluble scFv production

Mutant scFv genes from E1, E2, E3, E4, E6, E8, E13, E14, and E15 clones were amplified with KM148-KM253 and KM252-KM143 primers (Table 1), thus, changing TGA into TGG codon, as in original MA39 sequence, reassembled, and cloned in the antibody-expressing phagemid and transformed into HB2151 bacteria.

### Phage pool amplification and small scale scFv production

DH5αF' or TG1 bacterial cells infected with eluted phage were plated on agar-plates containing 100 μg/mL Ap (Sigma) and 1% glucose. Next day, a 50 μL aliquot of scrapped cells was incubated in 40 mL of LB (100 μg/mL Ap, 1% glucose) till OD_600 _= 0.2 with shaking at 37°C. Cells were spun down and resuspended in 40 mL of LB (100 μg/mL Ap), and then infected with M13K07 helper phage with a multiplicity of 20:1. Bacterial culture was incubated for a further 2 h at 37°C. Twenty μg/mL of Km (Sigma) were added to the bacterial culture, which was incubated on a shaker ON at 32°C. The rescued phages were PEG/NaCl-purified.

Supernatants of single phages were prepared as follows: infected individual TG1 colonies were inoculated in 150 μL 2×TY (100 μg/mL Ap, 1% glucose) in 96-well plates, incubated for 2 h at 37°C and superinfected with 10^9 ^pfu M13K07 helper phage in 25 μL 2×TY. After 30 min, plates were centrifuged at 1800 g for 10 min and bacterial pellet was resuspended in 200 μL 2×TY (100 μg/mL Ap, 20 μg/mL Km) and incubated on a shaker ON at 37°C.

For soluble scFv preparation, colonies were grown for 2 h at 37°C in 180 μL 2×TY (100 μg/mL Ap, 0.1% glucose) in 96-well plates and inducted with 50 μL 2×TY, containing 6 mM IPTG. On the following day, the plates were spun down at 1800 g for 10 min and the supernatants containing soluble scFvs were recovered and tested for specific CEA recognition.

### ELISA

Streptavidin-coated 96-well plates (Roche Diagnostics, Indianapolis, IN) were incubated ON at RT with 50 μL of biotinylated CEA (1 μg/mL) in PBS. After discarding CEA solution the plates were blocked with MPBS for 1 h at 37°C and rinsed with TPBS. One hundred μL of soluble scFvs in blocking buffer were added to each well and incubated for 1 h at RT. The plate was washed and incubated for 2 h with a freshly prepared mixture consisting of an anti-FLAG M2 (12.5 μg/mL, F3165, Sigma) and an anti-mouse HRP-conjugated antibody (22 μg/mL, Dako, Glostrup, Denmark). The immunoreaction was developed using 3,3^1^-5,5^1^-Tetramethylbenzidine (soluble BM blue POD substrate, Roche Diagnostics), stopped by adding 1 M sulfuric acid and read with an ELISA reader (Model 680 microplate reader, Biorad, Hercules, CA). Results were expressed as A = A(450 nm)-A(620 nm).

Alternatively, ELISA was performed by coating ELISA plate with 10 mg/mL of biotin-free CEA in 50 mM NaHCO3, pH 9.6. The phage antibody clones were tested using either of two secondary antibodies: an anti-FLAG HRP-conjugated (A8592, Sigma) or an anti-M13 HRP-conjugated secondary antibody (27-9421-01, Amersham Biosciences, Uppsala, Sweden).

Normalization of soluble scFv quantities was performed by coating ELISA plate with 2 μg/mL of a monoclonal anti-poly Histidine antibody (H1029, Sigma). Serial dilutions from 1/1000 to 1/27000 of each soluble scFv were applied, and ELISA was developed using an anti-FLAG HRP-conjugated secondary antibody.

### Soluble scFv purification

The MA39 and E8 antibody clones were cultured for large-scale scFv production. HB121 *E. coli *infected cells were cultured at 30°C in LB containing 100 μg/mL Ap and 0.1% glucose till OD_600 _= 0.5. After induction of antibody expression by adding 1 mM IPTG to culture, cells were incubated for another 3 h at 30°C. Periplasmic extracts were prepared by resuspending the bacterial pellet in 1/20 the original volume of 30 mM Tris, pH 7.0, 20% sucrose and 1 mM EDTA. After incubation for 20 min on ice, the debris was removed by centrifugation and supernatants (periplasmic fraction) were filtered (0.2 μm, Millipore, Bedford, MA). His-tagged scFv fragments were purified by immobilized metal affinity chromatography using Ni^2+^-nitriloacetic acid agarose (Qiagen, Madison, WI). ScFv fragments were eluted with 250 mM imidazole in PBS, dialyzed against PBS, tested for specific antigen recognition and stored at -80°C in small aliquots.

### Surface Plasmon Resonance (SPR)

Carcinoembryonic antigen (US Biological, Swampscott, MA) was immobilized on a CM5 sensor chip according to standard amine coupling procedure, using amine coupling kit (Biosense, Milan, Italy). Briefly, the carboxymethylated dextran-coated surface was activated by a 7-min injection of a solution containing 200 mM N-ethyl-N'dimethylaminopropylcarbodiimide (EDC) and 50 mM N-hydroxysuccinimide (NHS) followed by a 50-second injection of CEA (20 μg/mL in 10 mM sodium acetate, pH 3.7 adjusted with HCl). A continuous flow of HBS buffer (10 mM Hepes, 0.15 M NaCl, 3.4 mM EDTA and 0.005% surfactant P20, pH7.4) at 5 μL/min was maintained and capping of unreacted sites was achieved by injecting 1 M ethanolamine, pH 8.5. The final immobilization response was 421 Resonance Units (RU) equal to 0.421 ng/mm^2^. Blank surfaces were obtained in the same manner as kinetic surfaces, except that no protein was injected.

Sensorgrams for kinetic measurements were generated by the injection of soluble scFv (at 5 concentrations ranging from 62.5 nM to 4.5 μM) in HBS at a flow rate of 30 μL/min. Kinetic data were collected in duplicate for each antibody concentration. Association and dissociation times were 120 and 180 seconds, respectively. The chip was regenerated by injection of repeated pulses of 100 mM NaOH until the difference between the baselines before and after NaOH injection was less than 10 RU. Biosensor data were collected, modeled and fitted by means of BIAevaluation 3.1 software (Biacore, Uppsala, Sweden). The quality of the fitted data was evaluated by comparison between calculated and experimental curves (residual values) and by the magnitude of the chi^2 ^parameter.

### Specificity test

The CEACAM specificity test was performed by flow cytometry on BOSC23 cells transiently transfected with plasmids encoding for single CEACAM family members (Genovac, Freiburg, Germany). Briefly, 10^6 ^cells were seeded in 6-well plates and transfected with Lipofectamine 2000 (Invitrogen, Carlsbad, CA) according to the manufacturer's instructions. After ON incubation, the cells were collected, washed and resuspended in 200 μL PBS, 1% FCS at a concentration of 10^7 ^cells/mL. The flow cytometry reaction was carried out in 96-well plates in three steps using about 10^5 ^cells per well. Cell suspensions were first incubated with 25 μg/mL of primary scFv antibody; then, with 10 μg/mL of a secondary mouse anti-FLAG antibody (F3165, Sigma); and finally stained with a PE-conjugated anti-mouse antibody (550589, BD Pharmingen, Erembodegem, Belgium). Cells were incubated for 30 min at 4°C for each step, and washed with PBS containing 1% FCS. After final washing, cells were resuspended in PBS for acquisition and analysis using a FACSCalibur instrument with Cell Quest software (BD Biosciences, Erembodegem, Belgium). An irrelevant anti-glucose oxidase scFv antibody (enzyme not present in mammals) was used as negative control [13].

### CEA determination on cells

The expression of CEA on the surface of cells was quantified by using specific scFv in flow cytometry investigations on a consistent panel of human cell lines which include CCRF-CEM and JURKAT (acute lymphoblastic leukemia), GLC4 and H69 (small cell lung carcinoma), K-562 (chronic myelogenous leukemia), MCF7 (breast adenocarcinoma), RPMI8226 (myeloma), HT29 and LoVo (colorectal carcinoma), U937 (monocytic lymphoma), KB3.1 (cervical carcinoma), THP-1 (moncytic leukemia), HL-60 (promyelocytic leukemia), MV-4-11 (B myelomonocytic leukemia), Mel.1 and Mel.2 (melanoma cell lines prepared from primary melanoma specimens), and Mel.3 and Mel.4 (metastatic melanoma cell lines prepared from melanoma metastasis samples). HF, adult primary fibroblasts, (CD4+) PBMC subpopulation, purified monocytes (CD14+), granulocytes and neutrophils were also included in this study.

Adherent cells in exponential phase of growth were first trypsinized (we verified that CEA antigen is not sensitive to trypsin treatment), then collected, carefully washed in PBS 1% BSA and pelletted. About 2.5 × 10^5 ^cells were resuspended with 50 μL PBS, 1% BSA containing 5 μg/mL of primary scFv antibody and incubated for 1 h at RT. After several washings, cells were resuspended for 1 h at 4°C in a PBS, 1% BSA solution containing 10 μg/mL of an anti-FLAG mouse antibody (Sigma) and after washings, cells were incubated with 6 μg/mL of an FITC-goat anti-mouse IgG (#31541, Pierce, Rockford, IL) for 30 min at 4°C. An irrelevant antibody directed to glucose oxidase was used as negative control. After staining, the cell samples were washed, maintained at 4°C and immediately analyzed by FACScan (Becton, Dickinson and Company, Franklin Lakes, NJ) equipped with 15 nW argon laser. Fluorescence compensation was determined using samples stained with anti-glucose oxidase scFv and goat FITC-conjugated anti-mouse secondary antibody.

To study CEA distribution by scFv MA39, about 2 × 10^5 ^LoVo cells were spun down onto glass slides (Shandon, Pittsburgh, PA) or attached to poly-L-lysine-covered glass chamber slides (Labtek, Campbell, CA), fixed in 80% methanol solution for 10 min at 4°C. After soaking in TBS, the slides were treated for 10 min with peroxidase block solution (Dako EnVision System HRP, AEC), washed with RPMI, 10% FCS and incubated with 100 μL cell culture supernatant containing approximately 2.5 μg/mL scFv MAb MA39. Staining was performed by the peroxidase-antiperoxidase (PAP) method (Dako), counterstaining with Mayer hematoxyline (BDH, Milan, Italy).

### Western blot

Serial dilutions of purified CEA were fractionated by SDS/PAGE and transferred onto a nitrocellulose membrane. The membrane was blocked in PBS with 0.05% Tween 20 containing 5% non-fat dry milk. After washing, the membrane was incubated with soluble scFv antibody at 4°C ON, washed and then specific binding with scFv was detected by incubation for 1 h at 37°C with an anti-FLAG AP-conjugated antibody (A9469, Sigma) in 5% milk/PBS.

### DNA characterization of scFv antibodies

CEA-positive clones were analyzed by PCR amplification with primers KM143 and KM148. Agarose gel electrophoresis of PCR products confirmed integrity of scFv codifying inserts. Nucleic acid sequencing of the clones was carried out on an automated DNA sequencer Applied Biosystems Prism Dyes Terminator Sequencing Kit (PerkinElmer, Boston, MA) using KM143 and KM148 primers.

### Confocal laser scanning microscopy (CLSM) analysis

Melanoma metastatic cells (Mel.3) were grown on glass slides, and, after washing with PBS, incubated for 1 h at RT with 5 μg/μL of soluble scFv E8. At the end of incubation, the cells were washed with PBS and incubated for 30 min at 4°C with 2 μg/μL anti-FLAG (Sigma). Binding was detected with an FITC-conjugated anti-mouse antibody (Pierce) and then fixed with 4% paraformaldehyde for 5 min at RT. For CLSM analysis, coverslips were washed five times in PBS and mounted in buffered PBS-glycerine (1:1 v/v) solution; observations were performed using a Leica TCS SP2 spectral confocal microscopy equipped with argon-helium neon (Ar-HeNe) lasers and right filters for FITC fluorescence. Images were processed using LCS (Leica Microsystems, Heidelberg GmbH, Germany) and Photoshop (Adobe Systems Inc. Mountain View, CA) software programs.

### Immunohistochemistry

Immunohistochemistry studies were conducted on cryostatic sections of human lung cancer, melanoma and various normal tissues (TriStar Technology Group, LLC, Rockville, MD). Slides were processed according to standard protocols and binding revealed using Vectastain ABC (Vector Laboratories, Burlingame, CA). Briefly, the cryostatic tissue sections were fixed for 10 min with acetone at -20°C and endogenous peroxidase was blocked with 0.2% HCl in ethanol for 15 min. After two washes with TBS, slides were blocked with normal horse serum and then incubated for 2 h at RT (with 10 μg/mL of scFv E8). Slides were then washed and incubated for 1 h at RT with 10 μg/mL anti-FLAG monoclonal antibody (Sigma). After further washing, slides were incubated with avidin-biotin-peroxidase complex for 30 min. Finally, DAB substrate (Vector Laboratories) was added and the reaction was stopped after 5 min by washing in tap water. Counterstaining was performed with Mayer's hematoxylin (Vector Laboratories) for 10 seconds. Negative controls included slides incubated with secondary antibody alone. The staining intensity was evaluated by microscopic observation and graded from strong (+++) to no staining (-).

## Results

### Selection and specificity of MA39 scFv

In order to isolate phage-displayed specific antibodies, an aliquot of the human synthetic ETH-2 library containing approximately 1 × 10^12 ^TU was panned on biotinylated CEA, as described in Materials and Methods. After three rounds of selection, characterized by a progressive growth of output phage titer, several CEA-specific scFvs were isolated, since no cross-reactivity with streptavidin-coated plates was observed (data not shown). MA39, a phage antibody clone, one of the most reactive in ELISA, was cultured and quantities of purified scFv were produced for further investigation.

**Figure 2:**
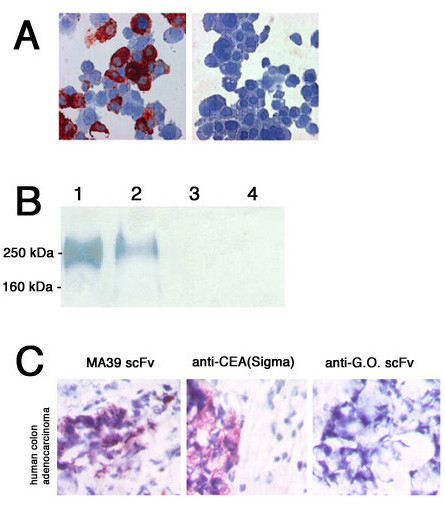
**Recognition of CEA by MA39 antibody**. A. Immunodetection of CEA in LoVo cells by MA39 scFv (left slide). The same slide preparation incubated with irrelevant anti-glucose oxidase (a protein not present in mammals) scFv antibody (anti-G.O., right slide). B. Biochemical patterns of the MA39 on purified CEA (lane 1) and LoVo cellular extract (lane 2). Lanes 3 and 4 show the reactivity of identical proteins with irrelevant anti-G.O. scFv. C. Staining of cryosections from human colon carcinoma xenograft in mouse. Commercially available anti-CEA antibody and irrelevant anti-G.O. antibodies were used as positive and negative controls, respectively.

Flow cytometry, biochemical and immunohistochemical studies indicate that scFv MA39 recognizes a genuine CEA epitope in its extracellular domain. In fact, MA39 binds to intact living human colon carcinoma LoVo cells (Figure 2A); in Western blot it recognizes a 200 kDa molecule of purified CEA antigen, and LoVo cellular extract (Figure 2B); it also stains human adenocarcinoma xenograft in mouse (Figure 2C).

### Maturation of anti-CEA scFv antibody

**Figure 3:**
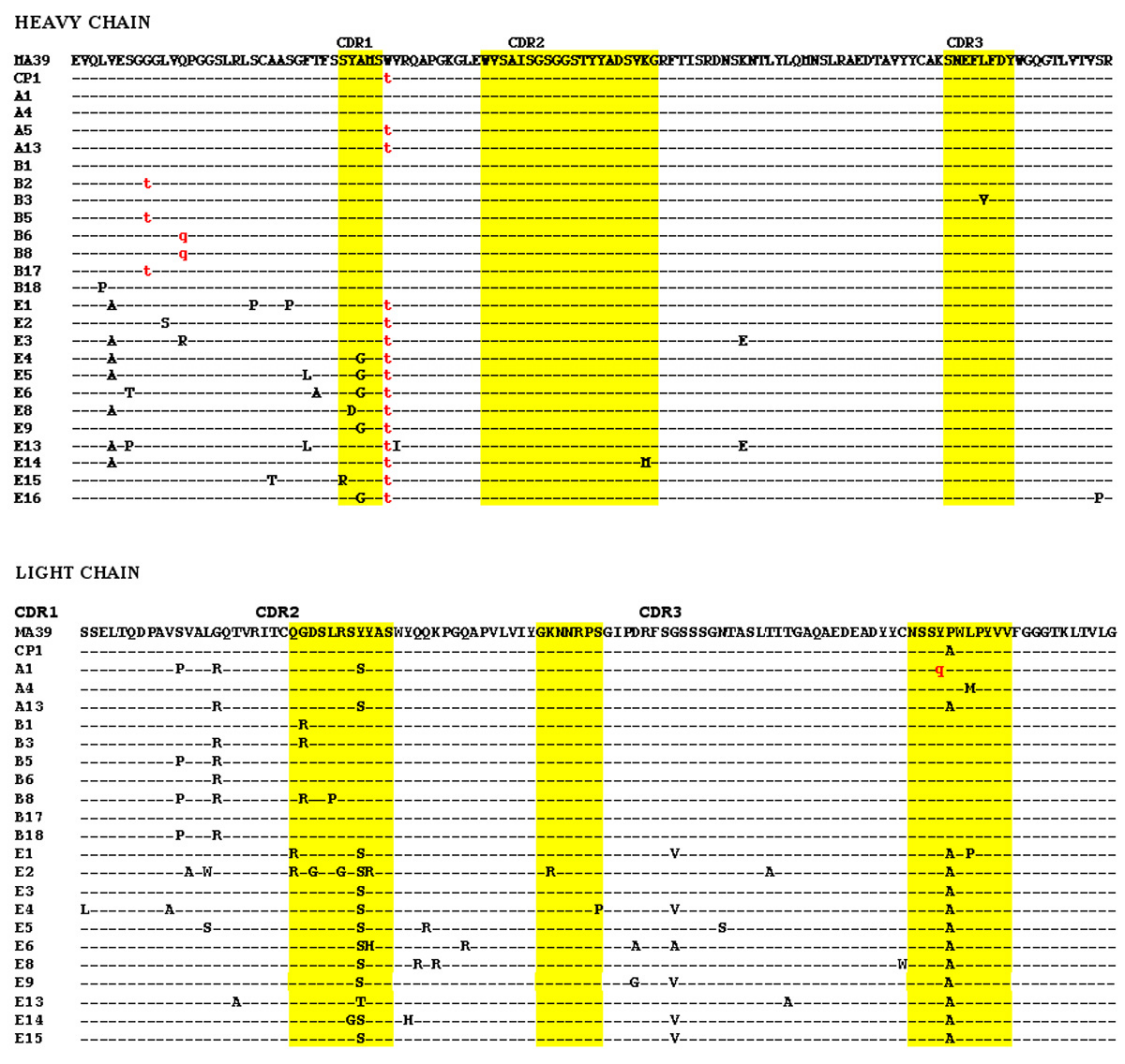
**Identified scFv antibody sequences**. Peptide sequences are reported in single-letter code. Amino acids in the mutated clones identical to parental MA39 are represented by a dash. CP1 – B18 and E1 – E16 groups of clones were isolated from first and second maturation libraries, respectively. Letters "q" and "t" correspond to glutamine and tryptophan amino acids in suppressed nonsense codons.

The first maturation library was constructed by introducing random mutations into CDR3 regions of heavy or light chains with low frequency, or by inclusion of 2–3 NNS random triplets at the 5' and 3' ends of heavy chain CDR3 of the MA39 scFv gene, as described in Materials and Methods (Figure 1). The library was affinity-selected three times against CEA protein. Phage random clones were prepared and tested in ELISA (data not shown). Clones with higher reactivity and the original MA39 scFv were sequenced to confirm mutation events. Figure 3 shows all sequences. There are only substituting mutations, but no insertions among the sequenced mutated genes. Unexpectedly, numerous selected phage clones (9 out of 12) harbor TGA, or TAG stop codon mutations. Bacterial strain DH5αF', used for phage amplification, suppresses TAG codon with glutamine aa residue. We find that this strain also suppresses the TGA codon. In order to test scFv antibody reactivity, ELISA was performed with phage supernatants and developed using anti-phage secondary antibodies. It excluded any influence of an incomplete antibody fragment participation in assay, confirming that full-length scFv antibodies fused with pIII protein in all these cases are displayed on the phage surface, implying suppression, probably with low efficiency, of TGA stop codons. Additionally, we also see the appearance of polar amino acids like arginine and serine, together with proline in amino-terminal part of light chain, in regions not undergoing the mutagenesis process.

Among first generation clones, CP1, which gained a mutation in CDR3 segment of light chain (P→A), was chosen for the next round of mutagenesis and maturation because of its reactivity. The CP1 gene contains a TGA codon within the heavy chain DNA fragment. We had no idea how common a phenomenon is the accumulation of nonsense codons that pull down the expression of phage antibodies during the selection process. It probably occurs when a high expression level of certain antibodies is toxic for bacteria. We decided to directly maturate the CP1 gene without TGA stop codon correction. The CP1 gene was mutated by error-prone PCR as described in Materials and Methods. This method permits the introduction of random mutations with efficiency up to 1% to 2%, using PCR amplification, performed with rising concentrations of Mg^2+^, lowering concentrations of dATP and by including dITP and Mn^2+ ^in reaction mixture [14,15]. Mutations were introduced in scFv gene regions corresponding to CDR1 and CDR2 zones of both chains (Figure 1). A second maturation library was selected twice against CEA protein, then single-stranded DNA was purified from phage pool and subjected to repeated error-prone mutagenesis; it was subsequently panned against target protein. A number of random clones (E1-E16) from last phage pool were tested in ELISA and sequenced.

### Production of soluble scFv antibodies

**Figure 4:**
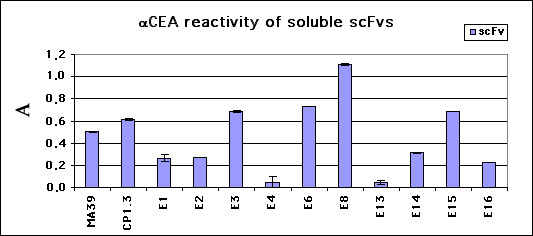
**ELISA reactivity of original and mutated anti-CEA antibodies**. Equal quantity of the soluble scFv antibodies were assayed on CEA-coated plates. Bound scFvs were developed by using anti-FLAG secondary antibody. Results are expressed as A = A_405_-A_620_. Data reported are the average values of two independent assays.

In order to analyze the relative reactivity of newly selected anti-CEA scFv mutants (the presence of TGA or TAG triplets in some phage clones with unknown suppression levels of stop codons prevents a correct analysis of antibody reactivity), several mutant scFv genes from E1, E2, E3, E4, E6, E8, E13, E14, E15 and E16 clones were amplified with KM148-KM253 and KM252-KM143 primers. The genes were reassembled and cloned into phagemid vector, thus reconstructing the original TGG codon as in parental MA39 gene. Soluble scFvs were produced in HB2151 bacterial strain, as described in Materials and Methods, and normalized by ELISA on anti-polyhistidine antibody-coated plates. Equal quantities of soluble antibodies were tested by ELISA for their capacity to bind CEA protein (Figure 4). The E8 scFv, having higher reactivity, was prepared together with MA39 in large amounts for measurement of their affinity and investigation of specificity.

### Comparison of original and affinity-maturated scFv antibodies

Plasmon resonance studies with BiacoreX provided quantitative measures of scFv-antigen binding and dissociation kinetics. The K_D _was calculated on the basis of the measured k_a _and k_d _rate constants by using a 1:1 Langmuir model for simple bimolecular interactions (Biaevaluation 3.0 software, Biacore International, Uppsala, Sweden). Table 2 reports the kinetic values of the parental and affinity-matured scFvs, determined on the CEA-coated chip. The original MA39 antibody has over 10 times lower affinity with the antigen, compared to E8, due to a slower association rate. The maturation process did not affect the dissociation rate.

**Figure 5:**
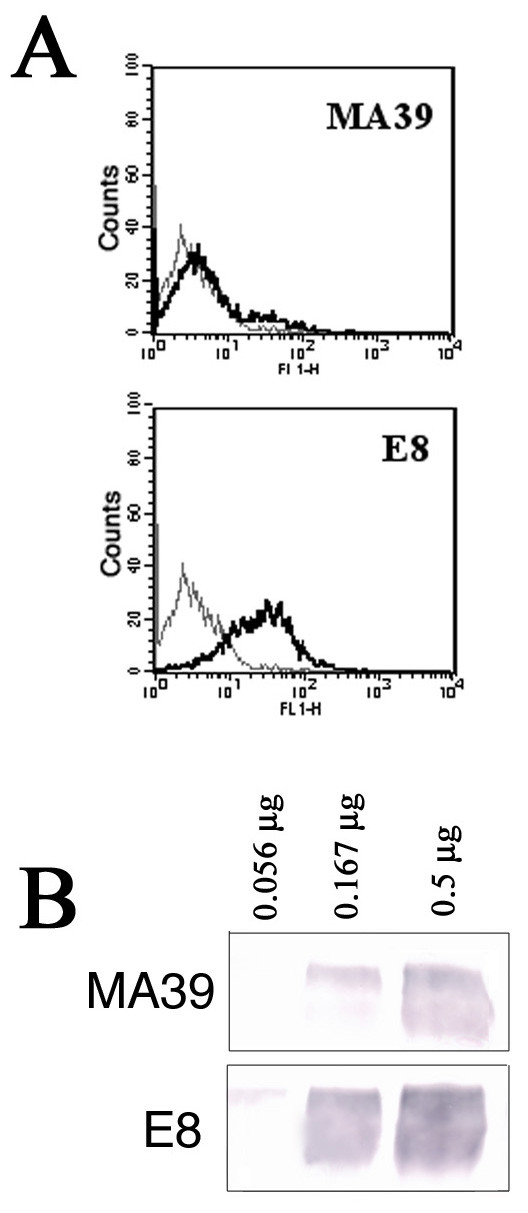
**Comparison between MA39 and E8 antibodies**. A. Recognition of metastatic melanoma cells expressing CEA by MA39 and maturated E8 scFv antibodies. Significant fluorescence shift indicates difference in affinity of two single-chain antibodies. B. Western blot with serial dilutions of CEA protein was stained by using original MA39 and maturated E8 scFv antibodies. The membranes were developed with secondary anti-FLAG AP-conjugated antibody.

A direct comparison of the CEA binding ability of MA39 and the maturated E8 scFvs was also assessed by flow cytometry analysis on a metastatic melanoma cell line (Mel.3). Results show that the increase in affinity is reflected in a significant fluorescent profile shift in the case of the E8 scFv (Figure 5A).

Western blot with serial dilutions of human CEA protein was developed with equal quantities of original and maturated antibodies (Figure 5B). The intensity of the developed color was increased when affinity-maturated scFv E8 was used for staining.

### Recognition of CEACAM proteins, human cells and human tumors by E8 scFv

**Figure 6:**
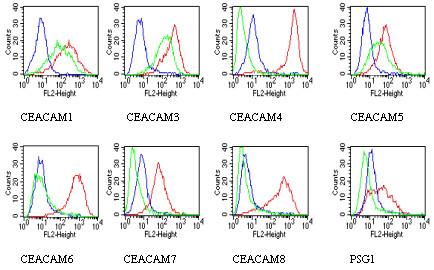
**Specificity test on transfectant cell lines**. Binding profiles of scFv E8 on BOSC23 cells transiently transfected with CEACAM gene family members are shown. Blue: anti-G.O. negative control antibody; Red: positive control mAb; Green: anti-CEA E8 scFv.

**Figure 7:**
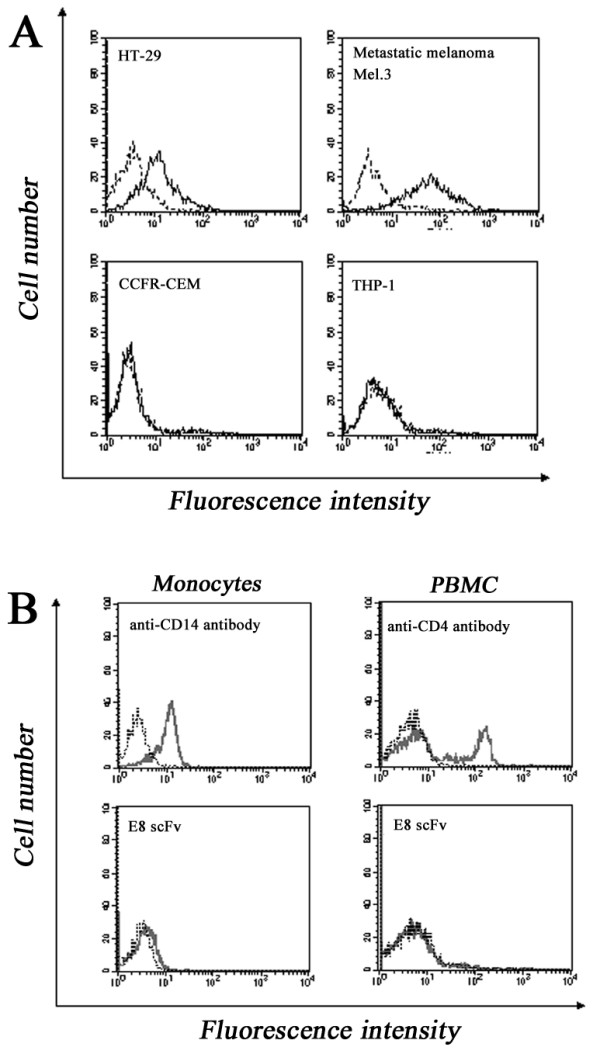
**Binding profiles of the scFv E8 on human cell types**. Dark line indicates level of reactivity of E8 and dashed line represents background binding level of the negative control scFv directed to glucose oxidase.

**Figure 8:**
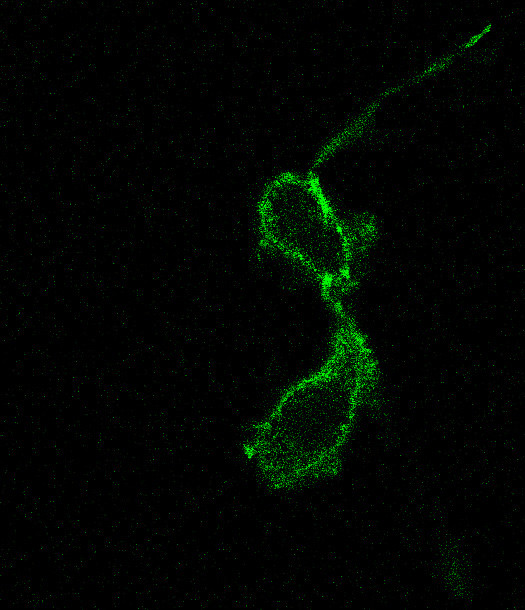
Spectral confocal microscopy of metastatic melanoma cells stained with E8 scFv antibody.

The affinity-maturated scFv E8 was tested for binding on eight different transiently transfected cell lines, each expressing a different member of the CEACAM protein family. According to this study the scFv antibody is reactive with CEACAM1 (BGP, CD66a), CEACAM3 (CGM1, CD66d) and CEACAM5 (CEA, CD66e) (Figure 6). This CEACAM recognition pattern of scFv E8 was also verified on a large panel of human cells which included 18 tumor cell lines, adult primary fibroblasts, PBMC subpopulations CD4 and CD14 and neutrophils (Table 3). As shown in Table 3 and partially in Figure 7A, scFv E8 strongly reacts with melanoma metastatic cells, and to a lesser extent, with colon carcinomas HT29 and LoVo, breast carcinoma MCF7, small cell lung carcinoma H69 and primary melanomas. In contrast, we see no binding with cervical carcinoma, several human leukemias, CD4 and CD14 subpopulations (Figure 7B) nor with human neutrophils. Figure 8 presents fluorescent staining of the metastatic melanoma cells.

**Figure 9:**
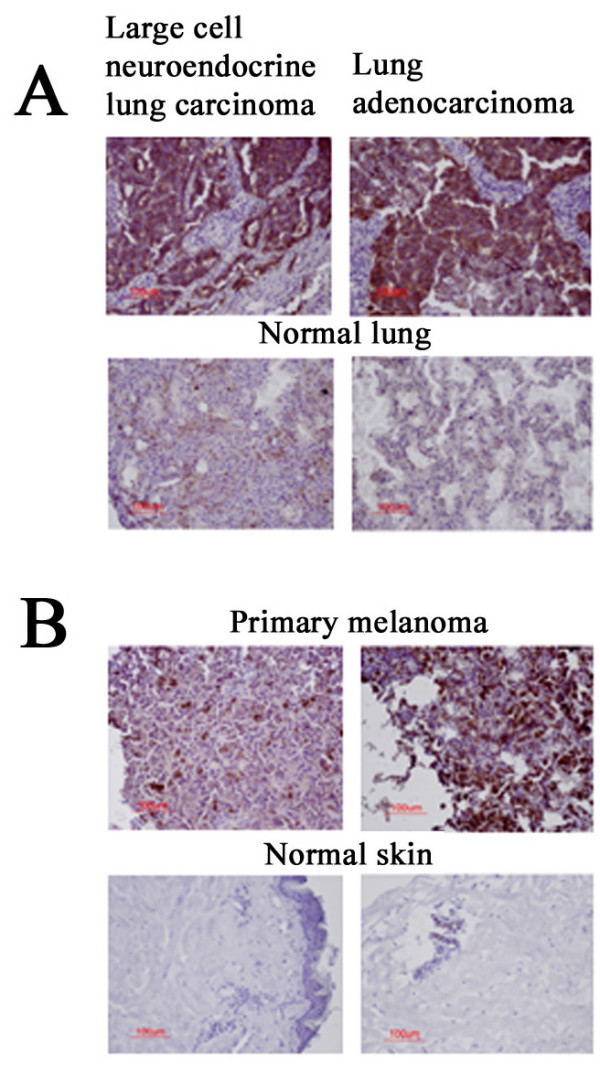
**Staining of cryosections from normal lung and lung carcinomas (A), normal skin and primary melanomas (B)**. Tumor tissues show strong staining with E8 scFv antibody.

We examined E8 scFv specificity by immunohistochemical staining on a large panel of samples obtained through cryostatic sectioning of various normal human tissues, human melanomas and lung cancers. E8 scFv specifically binds only tumor tissue. No unspecific staining was observed on a broad panel of human organs (Table 4, Figure 9). Weak background labeling of lung tissue was registered in one case out of nine tested. Negative control anti-G.O. antibody showed no staining (data not shown).

## Discussion

We were successful in selecting a primary single-chain variable antibody fragment against the human CEA protein from human synthetic ETH-2 scFv library. MA39 efficiently stained LoVo (human colon carcinoma) cells and human colon carcinoma xenograft in mouse, and detected a 200 kDa protein band on Western blot performed with LoVo protein extract, indicating the MA39 scFv as specific and sensitive reagent for immunochemical studies.

Additionally, we generated two successive maturation libraries to produce a second generation of scFv antibodies with higher affinity. In several cycles of *in vitro *mutagenesis, we introduced mutations with low frequency in CDR3 of heavy and light chains, and then in CDR1 and CDR2 regions of both chains. Analysis of mutated scFv genes, after two rounds of mutagenesis, revealed mutations that alter the primary structure of the antigen-binding sites and that also lie outside of CDR regions. These latter mutations appear to be important for protein folding, reducing the formation of aggregates, a known limiting process in the production of many recombinant proteins, including recombinant antibodies [16]. Indeed, gel filtration of MA39 and E8 scFv samples revealed a large fraction of protein aggregates in MA39 preparation (data not shown).

Sequence analysis of mutated clones after first round of mutagenesis revealed clear acquisition of stop codons during affinity selection, such as TAG, suppressed by DH5αF'-suppressing bacteria, and TGA, suppressed by natural suppression (suppression of nonsense mutation with native, not mutant tRNA [17-22]), where UGA triplet is decoded at a very low frequency by inserting tryptophan residue [23]. The acquisition of stop codons and mutations in non-mutagenized scFv gene regions probably indicates a strong selective pressure in bacteria for expression of the anti-CEA antibody.

CEA is an attractive target for immunotherapeutic purposes because of its expression profile, its role in tumor progression, and its immunogenicity. However, CEA belongs to the CD66 immunoglobulin superfamily that comprises highly homologous molecules expressed on several normal and transformed tissues and cells [24]. Flow cytometry investigations show that scFv E8 strongly reacts with a shared CEACAM1, 3 and 5 epitope. While the role of CEACAM5 has been extensively described in tumors [4], CEACAM1 and CEACAM3 are known to be expressed on human neutrophils, where their surface expression is increased following neutrophil activation by various stimuli [25,26]. Moreover, the cell adhesion molecule CEACAM1 is involved in intercellular adhesion and subsequent signal transduction events in a number of epithelia. CEACAM1 downregulation is evident in colorectal [27] and prostate [28] carcinomas; and the expression of CEACAM1 in prostate cancer cells suppresses their growth *in vivo *due to inhibition of tumor angiogenesis [29]. Nevertheless, CEACAM1 has recently received considerable interest as a cancer antigen target. Preclinical studies in melanomas show that expression of the cell adhesion molecule, CEACAM1, is an independent factor of the metastasis risk, with a predictive value superior to that of tumor thickness. Moreover, specific CEACAM1 gene mutation inhibits the invasive growth of melanomas, and treatment with anti-CEACAM monoclonal antibodies blocks CEACAM1-enhanced cell invasion and cell migration in a dose-dependent manner [30]. In fact, clinical studies in a 10-year follow-up indicate that expression of the cell adhesion molecule CEACAM1 in primary tumors in melanoma patients is associated with the subsequent development of metastatic disease [31]. CEACAM1 also appears to be a promising endothelial target for bladder cancer therapy; this molecule is involved in the switch from noninvasive and nonvascularized to invasive and vascularized bladder cancer [32]. Furthermore, CEACAM1, which is not expressed in the lung, has been identified in 2/3 of primary adenocarcinomas from this tissue and is considered a significant independent prognostic factor for survival [33].

Detailed analysis of E8 scFv specificity on normal tissue and cells, including skin samples, liver and neutrophils known for CEACAM1 expression, does not reveal any non-specific reactivity of the antibody, probably because of lower expression levels of CEACAM1 in normal tissue or non-stimulated neutrophils.

In comparison with many other anti-CEA antibodies, E8 is a completely human single-chain antibody of quite high affinity. To our knowledge, this is the first anti-CEA antibody isolated from a synthetic human scFv antibody library, thus indicating the recombinant antibody approach as an effective method for selection of potentially useful therapeutic antibodies. Due to its particular capacity to recognize CEACAM1 and CEACAM5 shared epitope, E8 recognizes a large panel of tumors (colon, breast and lung carcinomas and metastastic melanoma), and strongly binds tumors, such as lung carcinoma, that overexpress both the CEA proteins.

## Conclusion

The data reported and discussed in this paper indicate that this new immunoreagent meets several criteria for a potential anticancer compound: it is human, hence poorly or not immunogenic; it binds selectively and with good affinity to a CEA epitope expressed on human tumors, including melanoma, metastatic melanoma, lung, breast and colon carcinomas. It is particularly interesting that there is no reactivity in various normal human cells, including distinct classes of lymphocyte subpopulations and neutrophils, as well as a broad panel of human organs. While these findings propose scFv E8 for target therapy and diagnosis of solid tumors, further anticancer utilization of such a specific antibody, including direct inhibition of tumor cell proliferation and metastasization, needs to be carefully investigated.

## List of abbreviations used

aa – amino acid; EDC – N-ethyl-N'dimethylaminopropylcarbodiimide; NHS – N-hydroxysuccinimide; PE – phycoerythin; FCS – fetal calf serum; PFU – plaque-forming units; TU – transducing units

## Competing interests

EP, FP, AMA, VDA, AP, PV, GM, RDS, FF and OM receive salaries from Kenton s.r.l. or Sigma-Tau, the companies whose potential product was studied in the present work.

## Authors' contributions

EP and MF contributed in equal measure to this study. EP carried out scFv maturation library constructions, selection and characterization of improved scFv antibodies. MF carried out selection of the ETH-2 library against CEA and contributed to the genetic, molecular, biochemical and immunohistochemical characterization of scFvs. MLD and SB carried out the flow cytometry characterization of the MA39 scFv antibody and its affinity-maturated version, E8, using a large panel of primary and transformed human tumor cell lines. AA actively participated in phage antibody selection and carried out immunoassays and biochemical characterizations of scFvs against CEA. MC conceived and promoted the approach with the ETH-2 phage library to select specific scFv human antibodies against soluble CEA protein. Furthermore, MC participated in the design and coordination of the entire research project. PV and GM contributed to phage antibody selection and immunoassays. AMA, AP with supervision of FP performed flow cytometry of phage particles and soluble scFv antibodies, and Biacore analysis of scFvs. FP was also responsible for a specificity study. VD'A carried out immunohistochemical staining of normal and tumor tissue samples. OM planned molecular biology experiments for antibody maturation and teamed with FF and RDS in design and coordination of the entire project. All authors have read and approved the final version of the manuscript.

## Pre-publication history

The pre-publication history for this paper can be accessed here:



## Figures and Tables

**Table 1:** Oligonucleotide primers used for construction of mutant libraries for affinity maturation of anti-CEA MA39 scFv antibody. Wobble symbol N means G or A or T or C, symbol S means G or C. In underlined part of oligonucleotides KM144 and KM145, each base was replaced with mixture of G/A/T/C at a frequency of 10%.

KM144	5'-TGTGCGAAAAGTAATGAGTTTCTTTTTGACTACTGGGGC-3'
KM145	5'-TCCGCCGAATACCACATAGGGCAACCACGGATAAGAGGAGTTACAGTAATAGTCAGCC-3'
KM149	5'-GTATATTACTGTGCGAAANNSNNSNNSAGTAATGAGTTTCTTNNSNNSNNSTTTGACTACTGGGGCCAG-3'
KM150	5'-GTATATTACTGTGCGAAANNSNNSAGTAATGAGTTTCTTNNSNNSNNSTTTGACTACTGGGGCCAG-3'
KM151	5'-GTATATTACTGTGCGAAANNSNNSNNSAGTAATGAGTTTCTTNNSNNSTTTGACTACTGGGGCCAG-3'
KM152	5'-GTATATTACTGTGCGAAANNSNNSAGTAATGAGTTTCTTNNSNNSTTTGACTACTGGGGCCAG-3'
KM148	5'-CTATTGCCTACGGCAGCCGCTGGA-3'
KM157	5'-TTTCGCACAGTAATATACGG-3'
KM158	5'-TATGTGGTATTCGGCGGA-3'
KM143	5'-GTCATCGTCGGAATCGTCATCTGC-3'
KM169	5'-GGTGGTAGCACATACTACGCA-3'
KM170	5'-GGAATTGTCTCTGGAGATGGTGA-3'
KM171	5'-TCGTCTGAGCTGACTCAGGAC-3'
KM172	5'-GCATGTGATCCTGACTGTCTG-3'
KM173	5'-CTCCAGCTCAGGAAACACAGCTT-3'
KM174	5'-AGCCTCATCTTCCGCCTGAG-3'
KM252	5'-GAGCTGGGTCCGCCAGGCTCCAGGGAAGGGGC-3'
KM253	5'-GAGCTGGGTCCGCCAGGCTCCAGGGAAGGGGC-3'

**Table 2:** Kinetic values of parental and affinity-matured single chains

ScFv	k_on _(+/- SE)	k_off _(+/- SE)	K_D_
MA39	2.08E+04 (4.39E+02)	3.57E-03 (8.64E-05)	1.71E-07
E8	1.98E+05 (3.36E+03)	2.74E-03 (5.32E-05)	1.39E-08

**Table 3:** Flow cytometry determination of the binding level of the scFv clone, M39, and its affinity-matured variant, E8, on living/intact human cells. A. Stained cells: -, (0–5 %); +/-, (6–25 %); +, (26–50 %); ++, 51–75 %); +++, (76–100 %); B. Melanoma cells derived from characterized specimens of tumor patients; C. PBMC and specific cell subpopulations derived from blood of healthy donors. No differences were observed in MA39 or E8 reactivity using cells from different donors.

Cell type	Tissues	MA39	E8	Notes
HT-29	Colorectal adenocarcinoma	**+**	++	A
LoVo	Colorectal adenocarcinoma	+	++	
KB3.1	Cervical squamous cell carcinoma	-	-	
MCF7	Breast adenocarcinoma	+/-	+	
U937	Monocytic lymphoma	-	-	
Mel.1	Primary melanoma	+/-	+	B
Mel.2	Primary melanoma	+/-	+	
Mel.3	Metastatic melanoma	++	+++	
Mel.4	Metastatic melanoma	++	+++	
CCFR-CEM	T cell acute lymphoblastic leukemia	-	-	
H69	Small cell lung carcinoma	-	+/-	
Jurkat	T cell acute leukemia	-	-	
GLC4	Small cell lung carcinoma	-	-	
K-562	Chronic myelogenous leukemia	-	-	
RPMI8226	B lymphocyte myeloma	-	-	
MV-4-11	B myelomonocytic leukemia	-	-	
THP-1	Acute monocytic leukemia	-	-	
HL-60	Acute promyelocytic leukemia	-	-	
HF	Adult primary fibroblasts	-	-	
PBMC	total	-	-	C
CD4+		-	-	
CD14+		-	-	
Granulocytes		-	-	
Neutrophils		-	-	

**Table 4:** Immunohistochemical staining on cryostatic tissue sections

Tissue sample	Positively stained/Total tested samples
Melanoma	7/34
Normal skin	0/9
Lung cancer	15/35 (8 strong)
Normal lung	1/9 (few cells)
Normal tissues/organs	Negative on 29 different organs
